# The Safety of Continuous Infusion Propofol in Mechanically Ventilated Adults With Coronavirus Disease 2019

**DOI:** 10.1177/10600280211017315

**Published:** 2022-01

**Authors:** Corey J. Witenko, Audrey J. Littlefield, Sajjad Abedian, Anjile An, Philip S. Barie, Karen Berger

**Affiliations:** 1 NewYork-Presbyterian Hospital/Weill Cornell Medical Center, New York, New York, USA; 2 Weill Cornell Medicine, New York, NY, USA

**Keywords:** sedatives, pancreatitis, adverse drug reactions, respiratory failure, critical care

## Abstract

**Background:**

Propofol is commonly used to achieve ventilator synchrony in critically ill patients with coronavirus disease 2019 (COVID-19), yet its safety in this patient population is unknown.

**Objective:**

To evaluate the safety, in particular the incidence of hypertriglyceridemia, of continuous infusion propofol in patients with COVID-19.

**Methods:**

This was a retrospective study at 1 academic medical center and 1 affiliated teaching hospital in New York City. Adult, critically ill patients with COVID-19 who received continuous infusion propofol were included. Patients who received propofol for <12 hours, were transferred from an outside hospital while on mechanical ventilation, or did not have a triglyceride concentration obtained during the infusion were excluded.

**Results:**

A total of 252 patients were included. Hypertriglyceridemia (serum triglyceride concentration ≥ 400 mg/dL) occurred in 38.9% of patients after a median cumulative dose of 4307 mg (interquartile range [IQR], 2448-9431 mg). The median time to triglyceride elevation was 3.8 days (IQR, 1.9-9.1 days). In the multivariable regression analysis, obese patients had a significantly greater odds of hypertriglyceridemia (odds ratio = 1.87; 95% CI = 1.10, 3.21). There was no occurrence of acute pancreatitis. The incidence of possible propofol-related infusion syndrome was 3.2%.

**Conclusion and Relevance:**

Hypertriglyceridemia occurred frequently in patients with COVID-19 who received propofol but did not lead to acute pancreatitis. Elevated triglyceride concentrations occurred more often and at lower cumulative doses than previously reported in patients without COVID-19. Application of these data may aid in optimal monitoring for serious adverse effects of propofol in patients with COVID-19.

## Introduction

Coronavirus disease 2019 (COVID-19), caused by severe acute respiratory syndrome coronavirus 2 (SARS-CoV-2), was declared a pandemic by the World Health Organization in March 2020.^1^ In the ongoing global crisis, it has been estimated that 15% to 20% of cases require hospitalization and 3% to 5% require intensive care.^2^ Depending on definitions used and the degree of surging conditions, substantial worldwide variability has been reported with respect to the need for mechanical ventilation and outcomes of COVID-19–related critical illness. Data from Atlanta, Georgia at the onset of the pandemic suggested that 76% of critically ill patients required mechanical ventilation, with a mortality rate of 36%.^2^ By contrast, in 2 contemporaneous reports^3,4^ from metropolitan New York City, including the authors’ own institution,^3^ the need for critical care was estimated at 14%, the need for mechanical ventilation from 12% to 33%, and the mortality rate from 10% to 21%.

Respiratory failure from COVID-19 is often severe and protracted; patients requiring mechanical ventilation often require prolonged, deep sedation to optimize oxygenation and ventilation and facilitate ventilator synchrony. In the general critical care population, guidelines recommend use of nonbenzodiazepine sedatives, such as propofol or dexmedetomidine, as first-line remedies to improve short-term outcomes.^5,6^ However, patients with COVID-19 often require high doses of sedative medications for prolonged periods, and propofol is integral to achieving these goals.^7,8^

Propofol, an anesthetic agent formulated in a 10% lipid emulsion, has a favorable pharmacokinetic profile with a rapid onset and short duration of action.^5^ Possible adverse effects of propofol administration include respiratory depression, hypotension, hypertriglyceridemia, and propofol-related infusion syndrome (PRIS). Hypertriglyceridemia, in turn, can be associated with acute pancreatitis,^9,10^ which can be severe. The risk of acute pancreatitis is increased with triglyceride concentrations >500 mg/dL, and the risk is significantly higher with triglyceride concentrations >1000 mg/dL.^11^ As a result, many clinicians monitor triglyceride concentrations every 2 to 3 days during prolonged infusion and may switch to an alternative sedative agent if the concentration exceeds 400 to 500 mg/dL. These alternative agents are typically benzodiazepines, which have been associated with worse outcomes, such as increased delirium, longer duration of mechanical ventilation, and longer ICU and hospital lengths of stay.^6,12^ PRIS is a rare, potentially devastating complication associated with prolonged high doses (>83 µµg/kg/min for >48 hours), and has a reported mortality rate ranging from 18% to 48%.^13-15^ Clinical features of PRIS may include refractory bradycardia, metabolic acidosis, hyperkalemia, rhabdomyolysis, hyperlipidemia, acute kidney injury, or acute fatty liver.^16^ Finally, there is a potential risk of hypertriglyceridemia from propofol use in patients who develop secondary hemophagocytic lymphohistiocytosis (sHLH), a macrophage activation syndrome that, similar to COVID-19, can be associated with cytokine storm.^17^ Patients with sHLH may have elevated triglyceride concentrations at baseline.^18,19^

The objective of this study was to assess the safety of continuous infusion propofol in mechanically ventilated critically ill patients with COVID-19. We examined the incidences of hypertriglyceridemia, acute pancreatitis, PRIS, and propofol reduction or discontinuation caused by adverse effects. We hypothesized that hypertriglyceridemia is more prevalent among these patients and occurs at lower cumulative doses of the drug.

## Materials and Methods

This was a retrospective study of adult patients admitted to an intensive care unit (ICU) at an academic medical center, NewYork-Presbyterian Hospital/Weill Cornell Medical Center, and an affiliated teaching hospital, NewYork-Presbyterian/Lower Manhattan Hospital, between March 1, 2020, and April 30, 2020. Patients with a positive nasopharyngeal swab for SARS-CoV-2 by reverse transcriptase-polymerase chain reaction test and who received continuous infusion propofol for at least 12 hours were included. Patients were excluded if they received propofol for <12 hours, did not have a triglyceride concentration measured during propofol administration, or were transferred from an outside hospital already supported by mechanical ventilation. The study was approved by the Institutional Review Board of NewYork-Presbyterian Hospital/Weill Cornell Medical Center (20-05021995), with a waiver of informed consent. Patients were identified and relevant clinical parameters were obtained from electronic health records (EHRs) using the COVID-19 Institutional Data Repository (IDR). Data abstraction was completed on August 15, 2020. COVID IDR is a resource created and maintained by NewYork-Presbyterian Hospital and Weill Cornell Medicine, which brings together clinical and research information from disparate sources, including both ambulatory and inpatient EHR systems.^20^

The primary outcome was the incidence of hypertriglyceridemia defined as any triglyceride concentration of ≥400 mg/dL after propofol initiation. Additionally, we characterized the relationships among triglyceride concentrations and propofol dose and duration, the need for vasopressor therapy within 6 hours of propofol initiation, and the incidences of acute pancreatitis and possible PRIS. In patients with hypertriglyceridemia, the incidences of propofol infusion rate reduction and discontinuation were recorded, along with addition of adjunctive or alternative agents within 24 hours of dose reduction/discontinuation. A reduction in propofol rate was considered clinically meaningful if the reduction was by at least 20 µg/kg/min or at least 50% lower than the rate when hypertriglyceridemia was discovered. Adjunctive or alternative agents included chlordiazepoxide, diazepam, lorazepam, midazolam, dexmedetomidine, olanzapine, quetiapine, oxycodone, and methadone. Acute pancreatitis was defined as elevated pancreatic enzymes in serum (eg, amylase, lipase) and abdominal computed tomography with findings consistent with acute pancreatitis (eg, pancreatic edema or infarction, peripancreatic fluid collection[s]).^21-23^ Elevated pancreatic enzymes were defined as a serum amylase concentration ≥125 IU/L or serum lipase concentration ≥60 IU/L.

All patients with an elevated creatinine kinase (CK) concentration (≥5000 U/L) were evaluated for PRIS. Given that myositis is a clinical feature of COVID-19,^24^ the lack of diagnostic specificity, the nonspecific clinical presentation of PRIS, and lack of a standard definition,^14,15^ patients were considered to have “possible” PRIS if they had an elevated CK concentration and at least 2 of the following while receiving propofol: anion gap metabolic acidosis, serum lactate concentration ≥4 mmol/L, serum potassium concentration ≥5.5 mEq/L, acute bradyarrhythmia or cardiovascular collapse, acute kidney injury, or elevated concentrations of liver enzymes. Acute bradyarrhythmia was defined as a new drop in heart rate to less than 60 beats per minute not in sinus rhythm, and cardiovascular collapse included cardiac failure in the absence of cardiac disease. Acute kidney injury was defined as an elevation of serum creatinine greater than 50% from baseline or at least 0.3 mg/dL. Elevated liver enzymes were defined as an increase in concentrations to at least 3 times the upper limit of normal. Patients met the definition for possible PRIS if they had at least 2 of the listed criteria within 24 hours of elevated CK concentration. All laboratory monitoring, including triglyceride and CK concentrations, were obtained at the discretion of the provider.

Descriptive statistics were used to characterize the study sample with respect to demographic and clinical factors of interest. Continuous variables are represented as median (interquartile range [IQR]), and categorical variables are represented as n (%). Where appropriate, the χ^2^ test (or the Fisher exact test) and the Wilcoxon rank-sum test were used to examine the association between demographic/clinical factors of interest and the presence of hypertriglyceridemia. Statistically significant variables identified by univariate analysis (*P* < 0.10) were included in a multivariable logistic regression model to evaluate their independent effect on hypertriglyceridemia risk. Adjusted odds ratios (ORs) and 95% CIs for the risk/protective factors of interest were estimated from the multivariable model. All *P* values are 2 sided, with statistical significance evaluated at the 0.05 α level. All analyses were performed in R Version 4.0.2 (R Foundation for Statistical Computing, Vienna, Austria).

## Results

**Figure 1.**
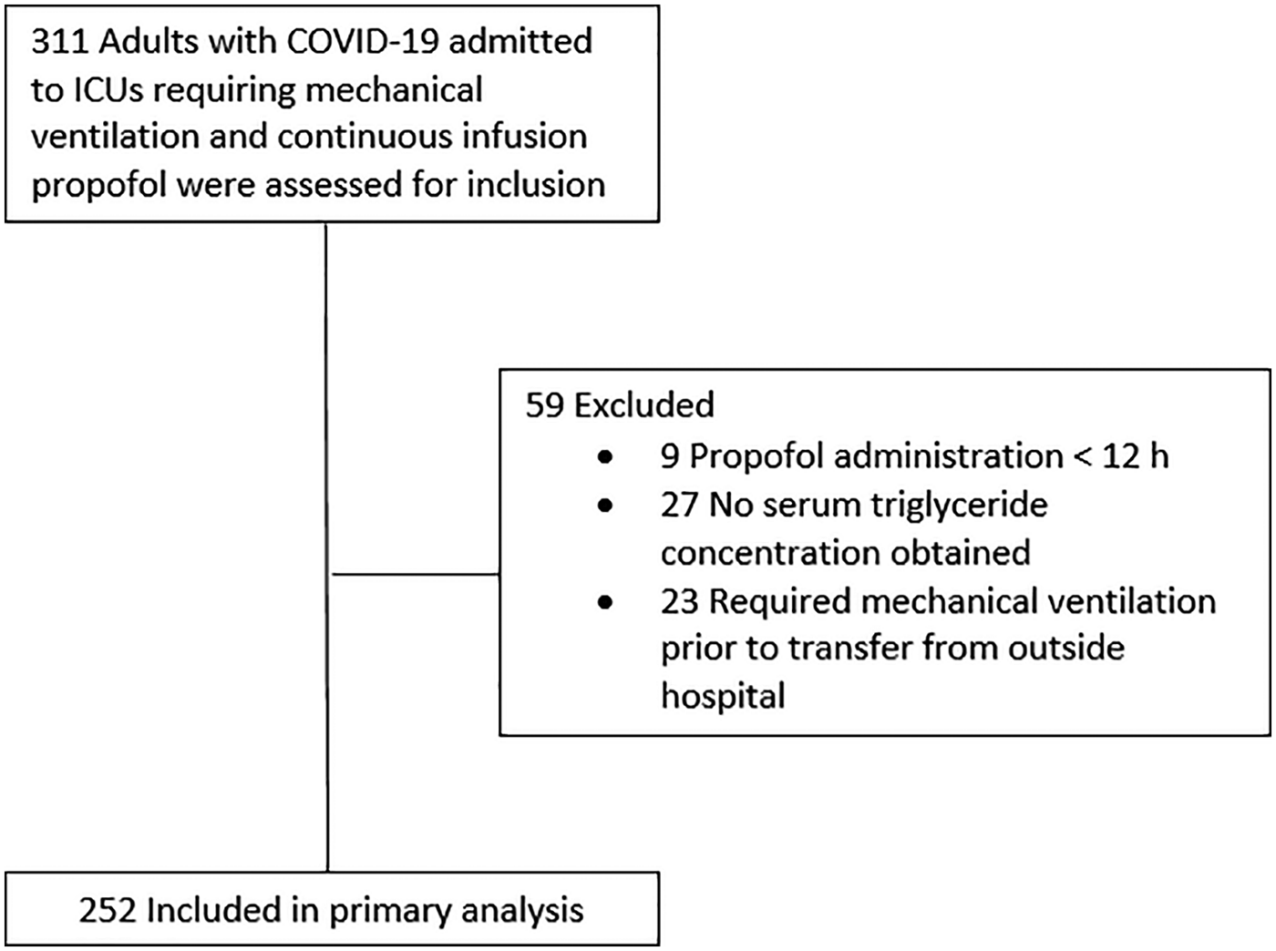
Patient flow diagram. Abbreviation: ICU, intensive care unit; COVID-19, coronavirus disease 19.

A total of 311 consecutive patients met inclusion criteria during the defined time period (Figure 1). In all, 59 patients were excluded as a result of either propofol administration for <12 hours, no serum triglyceride concentration measured, or transfer from an outside hospital while on mechanical ventilation. The 252 patients included in the primary analysis were mostly male (71.8%), with a median age of 67 years (IQR, 58-74 years; Table 1). No patient received parenteral nutrition support during propofol infusion. The cohort had high acuity, with a median Acute Physiology and Chronic Health Evaluation (APACHE) II score of 27 (IQR, 22-31)^25^ and Sequential Organ Failure Assessment score of 12 (IQR, 10-13).^26^ The median ICU and hospital lengths of stay were 33 days (IQR, 16.8-60.3 days) and 38 days (IQR, 20-62.2 days), respectively. The mortality rate was 32.5%, and the majority of survivors were discharged to a medical facility.

**Table 1.** Demographic and Baseline Characteristics.

Characteristic	n = 252
Age, years, median [IQR]	67 [58, 74]
Sex, n (%)	
Female	71 (28.2)
Male	181 (71.8)
Ethnicity, n (%)	
African American	22 (8.7)
Asian	47 (18.7)
White	69 (27.4)
Other/multiracial	49 (19.4)
Unknown	65 (25.8)
Weight, kg, median [IQR]	80.2 [69.0, 94.1]
BMI, n (%)^a^	
BMI < 30 kg/m^2^	151 (60.2)
BMI ≥ 30 kg/m^2^	100 (39.8)
Past medical history, n (%)	
Chronic obstructive pulmonary disease	28 (11.1)
Asthma	41 (16.3)
Coronary artery disease	65 (25.8)
Chronic congestive heart failure	11 (4.4)
Never smoker^b^	82 (59.9)
Diabetes type 1	7 (2.8)
Diabetes type 2	133 (52.8)
Hyperlipidemia	23 (9.1)
Hypertension	193 (76.6)
Chronic kidney disease	73 (29)
End-stage renal disease	34 (13.5)
Liver disease	9 (3.6)
APACHE II score, median [IQR]	27 [22, 31]
SOFA score, median [IQR]	12 [10, 13]
Vasopressor support (≥12 hours), n (%)	204 (81)
Mechanical ventilation duration, days, median [IQR]	21 [12, 45]
New renal replacement therapy, n (%)	20 (7.9)
Bacteremia, n (%)	53 (21)
Candidemia, n (%)	6 (2.4)
ICU length of stay, days, median [IQR]	33 [16.8, 60.3]
Hospital length of stay, days, median [IQR]^c^	38 [20, 62.2]
Discharge disposition, n (%)	
Hospital mortality	82 (32.5)
Discharge home	56 (22.2)
Facility	109 (43.3)
Still in hospital	5 (2.0)

Abbreviations: APACHE II, Acute Physiology and Chronic Health Evaluation II; BMI, body mass index; ICU, intensive care unit; IQR, interquartile range; SOFA, Sequential Organ Failure Assessment.

a n = 251 Patients with documented height.

b n = 137 Patients had a smoking status.

c n = 247 Patients because 5 were hospitalized at the time the date was finalized.

Hypertriglyceridemia occurred in 98 patients (38.9%) who received continuous infusion propofol (Table 2). The median time to elevated triglycerides was 3.8 days (IQR, 1.9-9.1 days), and the median total dose was 4307 mg (IQR, 2448-9432 mg; Table 3). Only 2.8% of patients had a serum triglyceride concentration ≥1000 mg/dL, which occurred at a median of 9.9 days (IQR, 7.9-19.7 days) from propofol initiation. The median infusion rate at the time hypertriglyceridemia was detected was 50 µg/kg/min (IQR, 40-60 µg/kg/min), and the incidences of decreasing or discontinuing the infusion within 24 hours were 35.7% and 16.3%, respectively. Adjunctive or alternative sedative agents were initiated in 26.5% of patients (eg, midazolam 46.1%, lorazepam 27%). Hypotension requiring vasopressor support within 6 hours after propofol initiation occurred in 11.1% of patients.

**Table 2.** Propofol Administration and Serum Triglyceride Concentrations.

Characteristic	n = 252
TG ≥ 400, mg/dL, n (%)	98 (38.9)
Time to TG ≥ 400 from propofol initiation, days, median [IQR]^a^	3.8 [1.9, 9.1]
TG ≥ 1000, mg/dL, n (%)	7 (2.8)
Time to TG ≥ 1000 from propofol initiation, days, median [IQR]^b^	9.9 [7.9, 19.7]
Patients with ≥2 TG concentrations^c^	
Initial TG after propofol initiation, median [IQR]	202 [138, 271]
Time to initial TG after propofol initiation, days, median [IQR]	1.3 [0.4, 2.5]
Maximum TG, mg/dL, median [IQR]	348 [239, 510]
Maximum increase in TG from initial TG, mg/dL, median [IQR]	115 [52, 271]
Time to maximum TG after propofol start, days, median [IQR]	8 [4.4, 14.3]
Any vasopressor initiation within 6 hours after propofol initiation, n (%)^d^	28 (11.1)
Norepinephrine	23 (9.1)
Vasopressin	6 (2.4)
Phenylephrine	26 (10.3)
Any pancreatic enzyme concentrations obtained, n (%)	103 (40.9)
Amylase obtained, n (%)	37 (14.7)
Lipase obtained, n (%)	103 (40.9)
Among those with pancreatic enzyme concentrations obtained	
Any elevated pancreatic enzyme concentration, n (%)^e^	61 (59.2)
Elevated amylase, n (%)^f^	15 (40.5)
Elevated lipase, n (%)^g^	60 (58.3)
Acute pancreatitis confirmed with radiographic imaging^h^	0
Possible PRIS, n (%)	8 (3.2)
No CK, n (%)	6 (2.4)

Abbreviations: CK, creatinine kinase; IQR, interquartile range; PRIS, propofol-related infusion syndrome; TG, triglycerides.

a n = 98.

b n = 7.

c n = 172, Excluding patients if initial triglyceride level was maximum.

d Epinephrine, dopamine, and angiotensin II were not initiated.

e n = 103.

f n = 37.

g n = 103.

h n = 12.

**Table 3.** Propofol Administration at the Time of Hypertriglyceridemia.^a^

Characteristic	n = 98
Cumulative propofol dose, mg, median [IQR]	4307 [2448, 9431]
Dose of propofol, µg/kg/min, median [IQR]	50 [40, 60]
Propofol discontinued within 24 hours, n (%)	16 (16.3)
Propofol decrease by ≥20 µg/kg/min or by 50% within 24 hours, n (%)	35 (35.7)
Propofol decreased by ≥20 µg/kg/min within 24 hours, n (%)	27 (27.6)
Decrease of propofol by 50% within 24 hours, n (%)	30 (30.6)
Addition of adjunctive/alternative agent within 24 hours, n (%)^b^	26 (26.5)
Midazolam	12 (46.1)
Lorazepam	7 (27)
Dexmedetomidine	2 (7.7)
Quetiapine	3 (11.5)
Olanzapine	1 (3.8)
Oxycodone	1 (3.8)

Abbreviation: IQR, interquartile range.

a All parameters pertain to the time of initial triglyceride ≥400 mg/dL.

b No patient had more than 1 agent initiated. Diazepam, chlordiazepoxide, and methadone were not initiated.

Comparing patients with or without hypertriglyceridemia, both groups had similar severity of illness, history of hyperlipidemia on admission, and hospital length of stay (Table 4), but there was a relationship between body mass index (BMI) and development of hypertriglyceridemia (*P* = 0.015), with a greater proportion of obese (BMI ≥ 30 kg/m^2^) patients in the hypertriglyceridemia group. Although likely statistical coincidence, there was a significant relationship between discharge disposition and development of hypertriglyceridemia (*P* = 0.042), with more patients discharged home in the hypertriglyceridemia group. Although cumulative propofol dose, duration, and time to peak triglyceride concentrations were similar, patients with hypertriglyceridemia had a longer duration of high-dose propofol administration (≥50 µg/kg/min; 3.9 vs 3.0 days, *P* = 0.036).

**Table 4.** Propofol Comparison With and Without Hypertriglyceridemia.

Characteristic	All patients, n = 252	Hypertriglyceridemia (TG ≥ 400), n = 98	No hypertriglyceridemia, n = 154	*P*
APACHE II, median [IQR]	27 [22, 31]	26 [21, 30]	27 [23, 31]	0.16
SOFA score, median [IQR]	12 [10, 13]	11.5 [10.2, 13.0]	12 [10, 13]	0.79
History of hyperlipidemia, n (%)	23 (9.1)	6 (6.1)	17 (11)	0.27
BMI, n (%)^a^				0.015
BMI < 30 kg/m^2^	151 (60.2)	49 (50)	102 (66.7)	
BMI ≥ 30 kg/m^2^	100 (39.8)	49 (50)	51 (33.3)	
Hospital length of stay, days, median [IQR]	38 [20, 62]	42.5 [23.0, 67.5]	35 [18, 59]	0.063
Discharge disposition, n (%)				0.042
Hospital mortality	82 (32.5)	30 (30.6)	52 (33.8)	
Discharge home	56 (22.2)	28 (28.6)	28 (18.2)	
Facility	109 (43.3)	36 (36.7)	73 (47.4)	
Still in hospital	5 (2)	4 (4.1)	1 (0.6)	
Cumulative propofol dose, mg, median [IQR]	10 777 [5801, 17 025]	11 730 [6442, 17 324]	9990 [5391, 16 732]	0.30
Duration of propofol infusion, days, median [IQR]	17.9 [9.1, 27.0]	18.5 [11.2, 31.3]	16.7 [8.5, 25.6]	0.071
Peak TG after propofol initiation, mg/dL, median [IQR]	336 [224, 502]	563 [466, 761]	244 [182, 318]	<0.001
Time to peak TG after propofol initiation, days, median [IQR]	5.8 [2.5, 10.8]	5.5 [2.6, 10.8]	6 [2.4, 10.7]	0.81
Duration of propofol dose ≥50 µg/kg/min, days, median [IQR]	3.4 [1.4, 6.8]^b^	3.9 [1.8, 7.6]^c^	3 [1.1, 6.4]^d^	0.036
Pancreatic enzyme concentrations, n (%)				
Amylase obtained	37 (14.6)	23 (23.5)	14 (9.1)	0.003
Amylase elevated	15 (40.5)^e^	11 (47.8)^f^	4 (28.6)^g^	0.42
Lipase obtained	103 (40.9)	52 (53.1)	51 (33.1)	0.003
Lipase elevated	60 (58.3)^h^	31 (59.6)^i^	29 (56.9)^j^	0.93
Acute pancreatitis	0	0	0	
Possible PRIS, n (%)	8 (3.2)	4 (4.1)	4 (2.6)	0.71
Tocilizumab, n (%)	41 (16.3)	20 (20.4)	21 (13.6)	0.21

Abbreviations: APACHE II, Acute Physiology and Chronic Health Evaluation II; BMI, body mass index; IQR, interquartile range; PRIS, propofol-related infusion syndrome; SOFA, Sequential Organ Failure Assessment; TG, triglycerides.

a n = 251 Patients with documented height.

b n = 237.

c n = 97.

d n = 140.

e n = 37.

f n = 23.

g n = 14.

h n = 103.

i n = 52.

j n = 5.

Pancreatic enzymes were not assessed routinely during propofol administration, but 61 (59.2%) patients had elevated concentrations when obtained. Providers checked pancreatic enzyme concentrations more frequently in those who developed hypertriglyceridemia (amylase: 23.5% vs 9.1%, *P* = 0.003; lipase: 53.1% vs 33.1%, *P* = 0.003). However, there was no difference in the incidence of elevated pancreatic enzyme concentrations. Although only 12 (19.7%) patients with elevated concentrations had imaging performed, there was no clinical or radiographic evidence of acute pancreatitis in any patient.

CK was checked frequently (at least once for 97.6% of patients), and 8 (3.2%) patients met our definition of possible PRIS, 4 of whom had hypertriglyceridemia. Six (75%) patients met at least 4 of our PRIS criteria in addition to CK ≥5000 U/L. The duration of propofol administration prior to CK elevation ranged from 2 to 17 days, and 5 (62.5%) patients received doses ≥40 µg/kg/min for >48 hours. Two patients (25%) died within 7 days of the identified CK elevation. There was no association of hypertriglyceridemia and possible PRIS.

A multivariable logistic regression model with BMI, duration of propofol ≥50 µg/kg/min, and duration of propofol infusion showed that obese patients independently had 1.87 times the odds of developing hypertriglyceridemia compared with nonobese patients, adjusting for propofol dose and duration of propofol dose ≥50 µg/kg/min (95% CI: 1.10, 3.21; Table 5).

**Table 5.** Multivariable Logistic Regression.

Characteristic	OR	95% CI	*P*
BMI ≥ 30 kg/m^2^	1.87	1.10, 3.21	0.022
Duration of propofol dose ≥50 µg/kg/min (d)	1.05	0.98, 1.12	0.18
Duration of propofol infusion (days)	1.01	0.99, 1.03	0.18

Abbreviations: BMI, body mass index; OR, odds ratio.

## Discussion

This is the largest study evaluating propofol use and safety in patients with COVID-19 and, to our knowledge, is also the largest study to date assessing the incidence of hypertriglyceridemia caused by propofol in a real-world setting. Given that patients with COVID-19 often have deeper sedation targets to facilitate comfort and ventilator synchrony during long-duration mechanical ventilation,^7^ it is important to elucidate the safety profile of propofol in this patient population.

Propofol is formulated in a phospholipid emulsion and may lead to hypertriglyceridemia with high doses or long-duration use.^5^ Elevated triglycerides in patients on propofol therapy have been associated with adverse sequelae, including acute pancreatitis.^9^ Although the evidence characterizing the relationship of hypertriglyceridemia and acute pancreatitis is scant, many clinicians use a triglyceride concentration of 400 to 500 mg/dL as a cutoff for using adjunctive or alternative sedative agents.^11^ Guidelines recommend monitoring triglyceride concentrations after 2 days of propofol infusion but not a threshold to prompt dose reduction or discontinuation.^27^ Interestingly, subsequent guidelines omit similar recommendations and do not provide further insight.^5,6^

A 2005 retrospective cohort study found that 18% of patients on propofol therapy had elevated triglycerides of ≥400 mg/dL, and 4% of patients had a concentration ≥1000 mg/dL.^9^ The incidence of hypertriglyceridemia-associated pancreatitis in the cohort was 1.9%.^9^ By comparison, the incidence of hypertriglyceridemia in our study was 38.9%, double the rate described by Devlin et al.^9^ Patients in our study were more critically ill (median APACHE II score, 27 vs 19) and had a longer ICU length of stay (33 vs 8.6 days). The propofol infusion rate upon hypertriglyceridemia diagnosis was similar at 50 µg/kg/min, but the cumulative dose at that time was substantially lower in our cohort (4307 vs 15 032 mg). Data on obesity, metabolic syndrome, and baseline triglyceride levels were not described in the study by Devlin et al, making it unclear if these contributed to the differences in hypertriglyceridemia seen in our cohort. Despite this, our data suggest that COVID-19 may lead to the development of hypertriglyceridemia more often and at lower cumulative doses, without an increased risk of acute pancreatitis.

A recent study at the same institution found that 27.9% of patients developed triglyceride concentrations ≥400 mg/dL while on propofol, with a 1.5% incidence of acute pancreatitis.^10^ In this study, patients developed hypertriglyceridemia after a median of 47 hours (IQR, 16.3-73.5 hours) and received a median cumulative propofol dose of 21 800 mg (IQR, 9300-32 400 mg). This contrasts with our study because patients developed hypertriglyceridemia after a median of 3.8 days and received a median cumulative propofol dose of 11 700 mg in our cohort. The higher incidence of hypertriglyceridemia at lower cumulative doses suggests that COVID-19 may have an impact on the metabolism and utilization of triglycerides.^8^

One small prospective observational study to date has compared propofol-associated hypertriglyceridemia in patients with and without COVID-19. Over a 7-day study period, the authors found that patients with COVID-19 had a higher incidence of triglyceride concentrations >500 mg/dL (33% [n = 27] vs 4.3% [n = 23]; *P* = 0.014).^8^ After correcting for differences in total propofol doses administered, COVID-19 was associated with an increased risk for developing hypertriglyceridemia (OR = 5.97; 95% CI: 1.16, 59.57; *P* = 0.031).^8^ Of note, patients with COVID-19 had a greater median BMI, baseline triglyceride concentration, and multiple higher serum inflammatory markers (eg, C-reactive protein, procalcitonin). This further supports our hypothesis and findings that patients with COVID-19 are at a higher risk of developing of hypertriglyceridemia.

Our study supports previously published evidence that propofol-associated acute pancreatitis is rare.^8-10^ However, in response to hypertriglyceridemia, providers in our study often lowered the dose or discontinued propofol altogether and added or transitioned to an alternative sedative, often a benzodiazepine, to facilitate propofol weaning. Guidelines recommend minimizing benzodiazepines to improve short-term outcomes such as risk of delirium and shorten the duration of mechanical ventilation and ICU length of stay.^6^ Midazolam in particular has a context-sensitive half-life and can accumulate during continuous infusion even absent any organ dysfunction.^28^ Therefore, transitioning from continuous infusion propofol to a benzodiazepine may have deleterious effects.

Although awareness of PRIS has increased,^29^ there is no consensus definition because it is not fully understood and exceedingly difficult to diagnose and characterize. Lower dosage and shorter-duration propofol infusions have been recommended for risk mitigation, although it is unclear whether PRIS is preventable.^29^ Clinical manifestations remain nonspecific, especially so in COVID-19, because components of PRIS such as acute kidney injury and elevated liver enzyme concentrations are observed commonly in this patient population.^30,31^ The estimated incidence of PRIS is 1.1%,^14^ and mortality rates range from 18% to 48%.^14,15^ The pathophysiology of PRIS is believed to be a result of mitochondrial dysfunction.^15^ One study found that screening for CK <5000 U/L eliminates the possibility for development of PRIS.^32^ Therefore, we selected a CK threshold of ≥5000 U/L as a prerequisite for possible PRIS in our study. EHRs of patients with CK ≥5000 U/L were rereviewed and demonstrated a 3.2% incidence of possible PRIS and, of those, a 7-day mortality rate of 25%. However, these data should not be conflated with the incidence and mortality of PRIS because several clinical features of this syndrome may also be present in severe COVID-19.

The triglyceride concentration alone should not be used as a monitoring parameter for PRIS because we could identify no correlation. This is consistent with a previous publication.^32^ Unfortunately, guidelines are silent as to recommendations for monitoring and treatment of PRIS.^6^ However, because of the prolonged, high doses of propofol administered to COVID-19 patients, providers should be mindful of the clinical manifestations of PRIS and the overlap with known COVID-19 manifestations and can monitor CK often to facilitate recognition and minimize morbidity.

There are other potential risks of using propofol in patients with COVID-19, specifically those who develop sHLH-like cytokine storm, because they may have elevated triglycerides at baseline.^18,19^ sHLH and COVID-19 critical illness can manifest similarly, possibly exacerbating the risk of propofol-related hypertriglyceridemia.^33^ A recent cross-sectional study demonstrated that high triglyceride concentrations were strong predictors of a severe course of COVID-19.^34^ The pathophysiology involves the overexpression of cytokines, and the inflammatory state produced during COVID-19 infection leads to significant changes in lipid metabolism, specifically affecting the lipoprotein lipase enzyme.^34^ This decreased enzyme activity results in decreased conversion of triglyceride-rich lipoproteins to low-density lipoprotein, which ultimately results in elevated triglyceride and decreased high-density lipoprotein concentrations.^34^ Another recent study described the dysregulation of lipid metabolism in patients with COVID-19.^35^ The authors found that patients with severe COVID-19 disease were more likely to have alterations in serum concentrations of lipids, including triglycerides, than those with mild or moderate disease.^35^ This has been described in other viral infections,^36^ and altered lipid metabolism ultimately leads to alterations in mitochondria homeostasis and energy production.^35^

One study^37^ that characterized patients with COVID-19 who received the interleukin-6 receptor antagonist tocilizumab^38^ for cytokine storm found that 29 patients (35.8%) experienced hypertriglyceridemia >500 mg/dL, and of those patients, 8 (27.6%) did not receive propofol. Tocilizumab-related hypertriglyceridemia has not been reported previously. In our cohort, only 16.3% of patients received tocilizumab, and there was no difference in the development of hypertriglyceridemia in these patients. When all these data are considered, it is evident that there are multiple mechanisms that may lead to elevated triglycerides in patients with severe COVID-19. Given the retrospective nature of this study, the exact causes of hypertriglyceridemia seen in patients with COVID-19 are uncertain and cannot be solely attributed to propofol administration.

Based on this study, we offer several recommendations and considerations regarding the safety of propofol use in COVID-19. First, monitor triglyceride concentrations within 2 to 3 days of propofol initiation and every 2 to 3 days, especially for obese patients and those receiving high-dose propofol infusions. Second, a triglyceride threshold of 400 to 500 mg/dL to discontinue propofol may be liberalized given the low risk of acute pancreatitis seen in this cohort as well as other published studies.^9,10^ Third, consider obtaining CK concentrations every 2 to 3 days to monitor for PRIS, especially for prolonged, high-dose propofol infusions.

This study has several limitations, including its retrospective nature, which render our observations speculative. Most of the data were pulled from an IDR, which raises the possibility that the results may be confounded by missing data. However, data elements in question were reviewed manually to ensure accuracy as far as possible. For example, missing data points may have caused the APACHE II scores to be underestimated. Although implausible, there is a possibility of falsely elevated triglyceride concentrations if any blood specimens were obtained via blood specimen collection utilizing the infusion port assigned to propofol. Such an event is unlikely to be documented, although no specimens were noted to be grossly lipemic. Unfortunately, because of the retrospective design and difficulty obtaining an accurate medication history, lipid pharmacotherapy was not reported in these data. It is our policy to maintain home medications while admitted unless contraindicated. In response to the pandemic, our health system expanded ICU capacity substantially into alternative physical locations that utilized health care personnel with minimal formal critical care training.^39-41^ Substantial effort was made to standardize protocols and communication among permanent and temporary ICUs.^39,42^ However, sedation practices, propofol utilization, and monitoring (eg, triglycerides, CK) varied among units, which may have led to selection bias as to laboratory monitoring. For example, 27 patients who were excluded from our analysis received propofol for a median 3.6 days but never had monitoring of triglyceride concentration. Further highlighting the need for education on the monitoring and toxicity of propofol, only 6 patients had a baseline triglyceride concentration drawn before propofol administration. Therefore, it is difficult to assess the direct impact of propofol on triglyceride concentrations. It is plausible that patients with severe COVID-19 have a higher risk for hypertriglyceridemia at baseline,^8,34,35^ and propofol administration may accentuate this risk. Moreover, only 12 (19.7%) patients with elevated pancreatic enzymes had follow-up imaging to assess the pancreas. Thus, the overall rate of acute pancreatitis may not be zero. Finally, some patients had propofol discontinued and restarted multiple times throughout their admission; quantified cumulative exposure may be inaccurate.

## Conclusion and Relevance

Hypertriglyceridemia occurred frequently in patients with COVID-19 critical illness who received continuous infusion propofol but did not lead to the development of acute pancreatitis. The only identified predisposing factor for hypertriglyceridemia was obesity. Elevated triglyceride concentrations occurred more often and at lower cumulative doses than reported previously in patients without COVID-19. PRIS was rare and challenging to diagnose but must be monitored carefully to aid in prompt recognition and intervention. Application of these data may aid in optimal monitoring for serious adverse effects of propofol used in patients with COVID-19.
